# MOOC Teaching Model of Basic Education Based on Fuzzy Decision Tree Algorithm

**DOI:** 10.1155/2022/3175028

**Published:** 2022-06-08

**Authors:** Zhang Yuanyuan

**Affiliations:** College of Education, Taiyuan Normal University, Taiyuan, Shanxi 030619, China

## Abstract

In recent years, the development of science and technology in China has greatly affected people's ways of entertainment. In the traditional industrial model, new industries and Internet industries represented by the Internet have emerged, and the Internet video business is an emerging business that has been gradually emerging in the Internet industry in recent years. Moreover, this new teaching method has been gradually noticed in simple education, such as MOOC, I want to self-study network, and Smart Tree, and other online learning websites have sprung up. At present, the epidemic environment makes people pay more attention to this convenient and wide range of online video education. Therefore, we need to evaluate this kind of online video teaching model from the effectiveness of this kind of method and the quality of user experience. This paper takes this as the starting point and chooses the earliest online video platform, MOOC, as the model to establish a set of perfect user experience quality evaluation methods suitable for domestic online video education mode. Considering the data source, the accuracy of the results, and other factors, we chose the industry-leading platform MOOC network as an example. Through the exploration of the MOOC teaching mode in basic education, a member experience evaluation model is established based on fuzzy decision tree algorithm. The experimental results show that the model has high accuracy and high reliability.

## 1. Introduction

With the popularity of online video software of Tencent, Youku, and Iqiyi, people gradually pay attention to the field of online video. The sudden epidemic has also made more and more people pay attention to the teaching model of online video education (Li et al.) [1]. Under the current epidemic situation, more and more schools choose to upload courses to online teaching platforms, which are funded by students to watch, study, and download. In addition, for some students who want to acquire professional knowledge in addition to school, some course videos taught by university teachers are the best way to learn, and video learning has become one of the ways and methods of national learning (Guand He) [2]. Therefore, in recent years, the Internet video business has gradually become an emerging business. More and more Internet companies begin to focus on and tend to the development of their video business. Development is bound to be accompanied by competition, and the competition of online video education is becoming more and more intense. The richness of video content and video quality determine the retention and experience of members (Farnaz et al.) [3]. However, live video education evolved from the traditional video industry, but it is quite different from the traditional new media business in terms of video content, video architecture, user needs, member audience, and so on (Hu et al.) [4]. Therefore, we cannot apply the traditional video model evaluation methods to the modern video education model, so we lack some evaluation and deep understanding of the current video education model. Although there are mature platforms abroad, such as courses, we need to consider the differences among China and foreign countries in international background and industrial development environment and user behavior. We can only learn from foreign research results, but we cannot take them as a guide (Vijay et al.) [5]. Therefore, how to establish a set of user experience quality evaluation methods for China's modern online video education model is very important and necessary. In view of this problem, we found through research that the MOOC platform is one of the earliest platforms for online video courses and video education in China. Compared with other platforms, there are hundreds of 985/211/double first-class universities on the MOOC platform, and their authority and richness of courses are far higher than other online video websites. Coupled with the influence of its years, the Muke platform can become the earliest representative model of online video education in China (fuzzy et al.) [6]. Therefore, taking Muke as an example, this paper discusses the user experience quality evaluation method of the Internet video education model.

The data cited below are from the real access data of the platform. Based on the collected data, we establish the user experience quality evaluation model of online Internet video learning through a fuzzy decision tree algorithm. For the collected data, we first clean the data and sort the collected initial text data into quantitative visual data, which is convenient for the next data analysis. After preprocessing the original data, the original data can show the login IP address and user agent field information to infer the type of access device. Finally, we get a series of data information such as member access video program information, member login information, and so on. On this basis, we use the mathematical algorithm, namely the fuzzy decision tree algorithm to statistically analyze the set to mine the characteristic laws of the data. For example, we can explore the distribution of video quality and the correlation between video quality and member retention rate, and lay a certain foundation for the next modeling work according to the mathematical characteristic laws we mine. Thus, an online video user experience quality evaluation model suitable for the current situation of Internet development in China is established, and the model is modeled based on a fuzzy number decision algorithm to evaluate and verify the accuracy and effectiveness of the model. Finally, the online video learning mode represented by the MOOC is evaluated by a fuzzy decision tree algorithm.

This paper establishes a set of perfect user experience quality evaluation methods suitable for domestic online video education mode. The research and innovation contributions include: through the exploration of MOOC teaching mode of basic education, a member experience evaluation model based on a fuzzy decision tree algorithm is established. The experimental results show that the model has high accuracy and high reliability. This set of evaluation teaching models is suitable for China's industrial development and combined with China's national conditions and provides a reference value.

## 2. Related Work

The evaluation of the online video learning mode is mainly to model and test the user experience under this mode. Therefore, this paper mainly explores the video viewing law and user experience of members using MOOC online video learning mode based on a fuzzy number decision algorithm. Among them, fuzzy data measurement algorithm is mainly used in video traffic measurement. Data analysis and sorting play an important role in the cleaning process (Sun) [7]. Firstly, the video measurement method is divided into the active and passive measurements. As the name suggests, the measured party actively obtains the specific browsing data of the measured party by sending data packets, so that the browsing records and interested video content of the measured party can be obtained quickly, conveniently, and directly. Therefore, the active measurement method has strong operability and simple, flexible, and direct operation (Teekaraman et al.) [8]. Passive measurement does not need to actively send data packets back to the user but directly pulls the required data from the network server through the browsing records of the network and the network characteristics on the statistical link of the background data packets. The alternative method does not need to send data. Therefore, it does not need to occupy the network transmission space and has little impact on the stability of the network. And because the data are directly pulled out in the background, the accuracy is also higher than the former (Fuzzy D et al. 2020) [9]. In the process of specific experimental design, we often choose different measurement methods according to the needs of specific conditions. After determining the way of data acquisition, we need to sort out and count the data to explore the law of member behavior behind the data. This law is mainly the law of the whole process after the member starts to execute the viewing behavior channel, and the process ends after the closing operation, including the relationship between the frequency of member switching video, viewing time, and the distribution of member resolution characteristics. Foreign research teams have conducted research on the frequency behavior of members' video conversions. This research analyzes the frequency distribution of member visits; that is, the frequency of collected member visits and online video teaching platform is drawn as zero, one, and more times (Raharja) [10]. According to the proportion of times of each frequency, it is found that the retention rate of members who use the PC terminal to initiate online video access requests is higher than that of members on the mobile terminal or iPad terminal, and the video conversion rate is lower in the viewing process, that is, less than 5% of users have switched videos in the viewing process (Jan et al.) [11]. The author of this article further clustered the video conversion time initiated by members and found that most of the video conversion time was when the frequency began to play (Mu et al.) [12]. Finally, according to the above collected data and the law of analysis from the data, we can do the user experience research and evaluation model of the online video education mode. The MOOC teaching evaluation model for basic education we proposed is an evaluation method that is in line with our current industrial development status and can effectively evaluate the online video teaching model, taking MOOC as an example. This method draws lessons from the existing evaluation models abroad to a certain extent. Florin Dobrain and others are researchers who have accessed data analyses through real online video platforms for the first time. They mainly explore the correlation between the member service scene and user participation (Shi and Huang) [13]. This is the first experiment using real scene data analyses, so the experimental results have certain reliability. However, the disadvantage is that the analysis conclusion of this study only depends on the actual data, and the specific reliability and accuracy have not been verified by relevant experiments. Subsequently, S. Shunmuga Krishnan et al. proposed to conduct research through quasi-experiment for the first time. They explored the relationship between video quality and member access behavior through experiments and finally found that there was a causal relationship between food quality and member access behavior (Khazali et al.) [14]. The authenticity and reliability of experimental results are more accurate and have higher credibility. As for the specific experimental methods, we can see the research on the relationship between food quality indicators and member participation in the visit process. Although s. shunmuga Krishnan et al. creatively improved the reliability of the experiment by using the experimental method in the research of the online video teaching model. At this time, the experiment mainly adopts qualitative measurement, which has a new overall impact on the correlation between the two in the experimental process, exploring the heterogeneity of scale and the rationality of the method. Subjective avoidance in the process of experimental design are not perfect and needs to be further discussed (Zhenget al.) [15].

## 3. Materials and Methods

**Figure 1:**
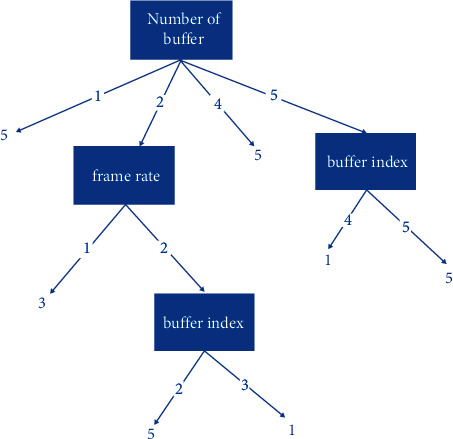
Example of a fuzzy decision tree generated by the algorithm.

The main purpose of this paper is to find a user experience evaluation scheme system suitable for the MOOC teaching mode of basic education. Firstly, the system needs to better fit the existing data on the MOOC teaching platform of basic education so that the evaluation system can become an effective proof of the credibility of the evaluation of member interview experience in the future. Therefore, the essence of the system is to reflect the correlation between video quality and member experience quality in the MOOC teaching mode of basic education and the optimal solution of linear relationship fitting. There is a certain balance between ordinary users and member users, which can neither make the ordinary video too simple nor let the member video monopolize. In the process of finding the optimal solution, it is inevitable to use data mining algorithm. Therefore, we first investigated several commonly used classification algorithms in data mining, such as the naive Bayes algorithm, decision tree algorithm, support vector machine, and the fuzzy decision tree algorithm. However, the Naive Bayes algorithm is suitable for scenes where data sets are independent of each other. However, the premise of this paper is that there is a correlation hypothesis between video quality and member access quality experience, so the classification effect of the model established by the naive Bayesian algorithm is not ideal. The decision tree algorithm is suitable for data sources that are discrete value sample sets, but the data sources and subsequent classifications we deal with regard to samples as continuous value sets, so the decision tree algorithm is not suitable for this paper. However, because the decision tree is a gradual algorithm based on dichotomy, the comprehensiveness and theory of the algorithm are relatively simple. For the deep mining algorithm suitable for the optimal solution in this paper, the decision tree algorithm can better fit the data results by removing the differences of data sources. Therefore, this paper adopts the fuzzy decision tree algorithm to solve this problem, which is more in line with the cognitive formula of data attribute characteristics and uncertainty, in theory. In the following practical experiments, we also take the decision tree algorithm as the comparison algorithm to evaluate the accuracy of the model. Because the remaining support vector machine algorithms are suitable for small-scale sample data mining problems, there are problems of long modeling times and complex algorithms in this paper, resulting in weak practical operability and eventually elimination. Therefore, this paper finally improves on the basis of the decision tree and adopts the fuzzy decision tree algorithm with more reliable and accurate results Figure 1.

Next, we collect and preprocess the data. The data we use are all from the actual access data of the MOOC network, and their content is only the log files in the process of member access, which do not involve the specific privacy of members. MOOC is an online video learning platform for basic education jointly developed by love course and Netease cloud classroom. It can be regarded as one of the first batches of the online video teaching platforms developed in China. Its platform has been settled in hundreds of universities, including 985/211/double first-class universities such as Tsinghua University, Peking University, Nankai University, and Fudan University. During the epidemic period, more and more schools choose to upload teaching videos to the MOOC platform, where students attend classes at home and complete homework and exams.

**Figure 2:**
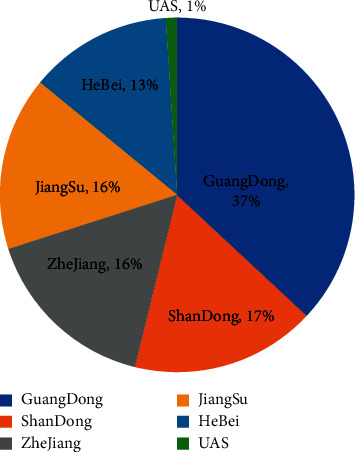
Member login area distribution statistics.

In addition, more and more people choose the MOOC platform for direct learning when they choose extracurricular knowledge other than their major, and nonstudents want to study courses on the university campus. Therefore, the MOOC platform has the amount of data that meet the needs of experimental research. In addition, the MOOC has the technical support of Internet head and huge database and video supports network TV, IP mobile phone IPad, PC, and other playback platforms, which also provide basic data support for us to study the behavior of members accessed by different terminals. We collect the key information from the collected original data, clean the duplicate information and fill in the vacancy information, identify the access behavior, determine the key video quality, and calculate the address and location of member access. Finally, the data result we sorted out is the collection of behavior records of the MOOC online video education platform Figure 2. Each record includes three parts: video information accessed by members, identity information of members, and specific behavior process information.

Next, we will rely on the fuzzy decision tree algorithm to establish an evaluation model that reflects the correlation between the video quality visited by members and the actual experience quality of members. The following is the theoretical basis of the fuzzy decision tree algorithm based on the classification fuzziness. First, we define the size of fuzzy set *A*, as shown in the following formula:

(1) $$\begin{array}{c} M\left(A\right)=\sum_{u\in U}\mu_{A}\left(\mu \right). \end{array}$$

When *A* represents a fuzzy set on the full data set U, it is included in the function *μ*_*A*_. When *U* is a discrete set, the fuzziness of fuzzy set U is defined in the following formula:

(2) $$\begin{array}{c} M_{V}\left(A\right)=-\frac{1}{m}\sum_{i=1}^{m}\left(\mu_{i}\ln\;\mu_{i}+\left(1-\mu_{i}\right)\ln\left(1-\mu_{i}\right)\right). \end{array}$$

For the discrete set *X*, according to the fuzzy decision tree algorithm, if there is a normalized distribution of variable *Y* on its set, the fuzziness of variable *Y* is defined as the following formula:

(3) $$\begin{array}{c} E_{a}\left(Y\right)=g\left(\pi \right)=\sum_{i=1}^{n}\left(\pi^{•}-\pi_{i+1}^{•}\right)\ln\;i. \end{array}$$

It can be deduced from the above definition that when the variable *Y* can only take one value, the value of the fuzziness set is 0, which means that the variable *Y* does not have fuzziness at this time. When the possibility that variable *Y* can take any value in set *X* is 1, *Y* has the greatest fuzziness. When the numerical attribute is continuous, variable *A* can form a set containing *S* discrete semantics after fuzzy processing, in which each semantic item is a fuzzy set. In order to measure the fuzziness of *A* the membership function value of any variable in the fuzzy set is set *u*, and the fuzziness possibility distribution of continuous attribute *A* can be obtained. First, normalize the probability distribution of set *u*, as shown in the following formula:

(4) $$\begin{array}{c} \pi_{T_{\delta }}\left(u_{i}\right)=\frac{\mu_{T_{\delta }}\left(u_{i}\right)}{\underset{1\leq j\leq S}{\max}\left\{\mu_{T_{\delta }}\left(u_{i}\right)\right\}},\;s=1,2,3,\ldots ,S,\\ E_{\alpha }\left(A\left(u_{i}\right)\right)=g\left(\pi_{T}\left(u_{i}\right)\right),\\ E_{\alpha }\left(A\right)=\frac{1}{m}\sum_{i=1}^{m}E_{\alpha }\left(A\left(u_{i}\right)\right). \end{array}$$

From the above, the fuzziness measurement formula of continuous variable *A* can be obtained, and the result can also be applied to the measurement of fuzziness of classification results. Next, we discuss the fuzziness rule and its confidence level. In the fuzziness rule, two conditional fuzzy sets *A* and *B* have been defined, and it is assumed that there is a corresponding relationship between them. Therefore, we need to define the authenticity of the rule by using the concept of confidence level, expressed in *S*(*A*, *B*). See formula (5) for the specific calculation formula. At this time, the category possibility judgment formula of attribute variables is the following formula:

(5) $$\begin{array}{c} S\left(A,B\right)=\frac{M\left(A\cap B\right)}{M\left(A\right)} \end{array}$$

(6) $$\begin{array}{c} \pi \left(C_{t}|E\right)=\frac{S\left(E,C_{i}\right)}{\max S\left(E,C_{i}\right)},\;j=1,2,3,...,L. \end{array}$$

Combining the fuzziness measurement formula with the confidence level, we can redefine the classification fuzziness measurement formula. See the following formula: for details:

(7) $$\begin{array}{c} G\left(E\right)=g\left(\pi \left(C_{t}|E\right)\right). \end{array}$$

At this time, assuming that the value of variable *A* on set *u* is *F* and the fuzzy semantic item set corresponding to variable *B* is *p*, the calculation formula of fuzzy classification and division of the correlation between variables *A* and *B* in the fuzzy decision tree algorithm rules can be seen in the following formula:

(8) $$\begin{array}{c} G\left(P|F\right)=\sum_{t=1}^{k}w\left(B_{t}|F\right)G\left(B_{t}|F\right), \end{array}$$

where *w*(*B*_*t*_*|F*) represents the size of fuzzy set *F*, see formula (9) for specific calculation method, and see formula (10) for fuzzy evidence at this time:

(9) $$\begin{array}{c} w\left(B_{t}|F\right)=\frac{M\left(B_{t}\cap F\right)}{\sum_{j=1}^{k}M\left(B_{j}\cap F\right)}, \end{array}$$

(10) $$\begin{array}{c} \mu_{E_{\alpha }}\left(u\right)=\left\{\begin{array}{cc} \mu_{E}\left(u\right), & \mu_{E}\left(u\right)\geq \alpha ,\\ 0, & \mu_{E}\left(u\right)<\alpha . \end{array}\right. \end{array}$$

Finally, we bring the preprocessed data into the semantic item membership function to complete the data fuzzification, which obtains the parameters by using the Kohonen feature mapping algorithm and realizes the transformation from the fuzzy decision tree algorithm to fuzzy rules. The induction process of a fuzzy decision tree consists of the following steps: (1) data preprocessing; (2) induction and establishment of decision tree; (3) The obtained fuzzy decision tree is transformed into a set of fuzzy rules; and (4) The obtained fuzzy rules are applied to classification. The membership function of semantic items is shown in the following formulas (11)–(13):

(11) $$\begin{array}{c} \mu_{T_{k}}\left(x\right)=\left\{\begin{array}{cc} 1, & x\geq m_{k},\\ \frac{\left(x-m_{k-1}\right)}{\left(m_{k}-m_{k-1}\right)}, & m_{k-1}<x<m_{k},\\ 0, & x\leq m_{k-1}, \end{array}\right. \end{array}$$

(12) $$\begin{array}{c} \mu_{T_{1}}\left(x\right)=\left(\begin{array}{cc} 1, & x\leq m_{1}\\ \frac{\left(m_{2}-x\right)}{\left(m_{2}-m_{1}\right)}, & m_{1}<x<m_{2}\\ 0, & x\geq m_{2}, \end{array}\right. \end{array}$$

(13) $$\begin{array}{c} \mu_{T_{i}}\left(x\right)=\left\{\begin{array}{cc} \frac{\left(m_{i+1}-x\right)}{\left(m_{i+1}-m_{i}\right)}, & m_{i}<x<m_{i+1},\\ \frac{\left(x-m_{i-1}\right)}{\left(m_{i}-m_{i-1}\right)}, & m_{i-1}<x<m_{i},\\ 0, & x\geq m_{i+1}\cup x\leq m_{i-1.} \end{array}\right. \end{array}$$

**Figure 3:**
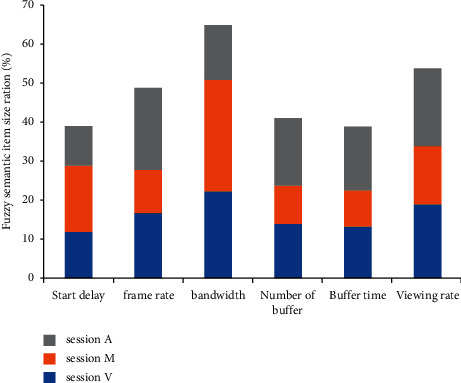
Different sets of video viewing index size proportional relationship.

In order to verify the member experience quality evaluation of the MOOC teaching model of basic education based on fuzzy decision tree algorithm, this study designs the following experiments to evaluate the model. Firstly, the data mining classification accuracy of fuzzy decision tree is calculated by the tenfold cross validation method. In order to prove the accuracy of the fuzzy decision tree algorithm, in addition to using the fuzzy decision tree algorithm to process data, we also use the decision tree algorithm to fully prove the accuracy of the algorithm. We randomly selected three different data sets, session *V*, session *M*, and session *A*. Where session *V* represents all access records under a video, session *M* represents access records of different device types, and session a represents the video access records in different regions. By changing the parameters, observe the changing trend and fitting accuracy of the fuzzy decision tree model on the three different data sets of session *V*, session *M*, and session *A*, and compare the dependent variables under different conditions, so as to judge the impact of the quality of the video, the region of the members, and the equipment of the members on the accuracy of the prediction results of the model Figure 3.

**Figure 4:**
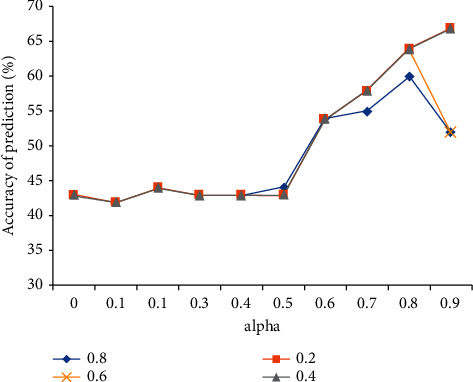
Fuzzy decision tree model predicts accuracy when parameters are different.

After obtaining the preliminary verification results, we need to consider its reliability. Therefore, we continue to compare at different levels: significance level a and confidence level B. When a and B take different values respectively, the prediction accuracy of fuzzy decision tree models session a, session m, and session V changes. The results are shown in Figure 4. Then, taking conversation a as an example, we show different significance levels a and confidence levels B. When the accuracy changes, we can see that whether the value of B is any of 0.2 to 0.8, the session set prediction accuracy remains at about 43% without obvious fluctuations. When the value of a is 0.5, it is different from B. when the value of a is 0.8, the prediction accuracy of the session begins to differ at the level of B. The maximum prediction accuracy of session a is 44%. However, compared with Class A and class B, the prediction accuracy under Class A and class B still has no significant difference. When the value of a is 0.6, the prediction accuracy of level B for level a sessions is higher. However, when the b value is 0.8, the prediction accuracy of level a sessions is lower than that of level B sessions. The prediction accuracy of level a sessions is 0.2-0.6. When the value of a is 0.9 and the value of B is 0.2, the prediction accuracy of session a is the highest, which is 67%. When e of a is 0.1 and B is 0.2, the prediction accuracy is the lowest, which is 42%. The predicted change law of session a is basically the same as that of session C.

## 4. Result Analysis and Discussion

**Figure 5:**
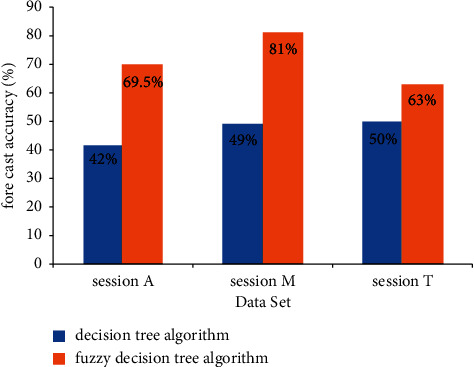
Comparison of prediction accuracy between fuzzy decision tree and decision tree model.

In the MOOC teaching model of inquiry basic education, in the experience quality evaluation system of members for online video teaching, if only the technical needs of data mining are considered, the decision tree algorithm can be met in the commonly used data mining technology. However, because the decision tree algorithm is only used for the processing of discrete attribute data sources in practice, it has some limitations on the processing of continuous variables, such as access time. Therefore, based on the existing data mining technology, this paper proposes a fuzzy decision tree algorithm. The specific advantages and disadvantages of the algorithm and the decision tree algorithm have been compared in detail at the beginning of the third part, so it is not repeated here. Only the comparison results of the prediction accuracy of the decision tree algorithm and the fuzzy decision tree algorithm on the three sets of session *A*, session *M,* and session *T* are presented. The specific results are shown in Figure 5. From the figure, we can see that for different data sets session *A*, session *M*, and session *T*, the model prediction accuracy based on fuzzy decision tree algorithm is always higher than that based on decision tree algorithm, which further verifies the rationality of our algorithm. In addition, among different sets, the model prediction accuracy of session *M* based on the fuzzy decision tree algorithm is the highest, reaching about 81%, while the model prediction accuracy of session *T* based on the fuzzy decision tree algorithm is the lowest, about 63%. It is preliminarily speculated that this is due to the different degree of analysis of the video quality capture of members' access and the capture accuracy of members' access to geographical locations. This problem can be further explored in subsequent research.

**Figure 6:**
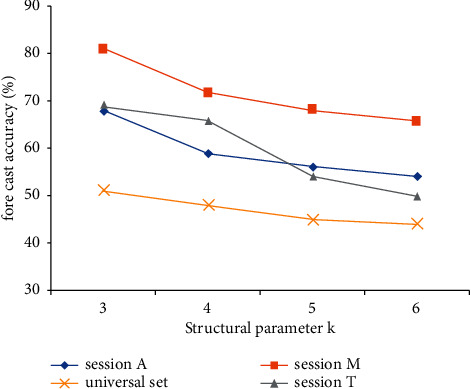
Comparison of model prediction accuracy under different subsets and different *K* values.

In addition, we notice that the classification accuracy of fuzzy decision tree is also different on different subsets and complete sets. It can be found from Figure 6 that the prediction accuracy of the accuracy prediction model on subsets session *A*, session *M*, and session *T* far exceeds that on the full set *S*. This result shows that the experience of members accessing the online video teaching platform is greatly related to the video content, region, and equipment accessed by members. The gap between each factor is large, and there is interaction. We speculate that the reasons are as follows: Firstly, it is related to the members' own interests. The interest in video affects the members' tolerance of video. Secondly, it will be affected by the background, cultural habits, and local policies of the member's location. For example, there are great differences in the learning methods between the first tier cities and the second and third tier cities. Due to development constraints, the second and third tier cities may not understand the online platform channels, so the visit will also be affected. Or if the local requires students to teach on average, the local visit volume will also be greatly affected. In addition, using different devices to access the MOOC platform also represents the current state of members to a certain extent, so it will affect the accuracy of the evaluation model. Starting from practical reasons and exploring from the perspective of fine granularity, we can still find out other objective influencing factors. Due to the length of this paper, we will not explore in detail.

## 5. Conclusion

The main goal of this paper is to establish a set of evaluation teaching models suitable for the development of China's industry and combined with China's national conditions. Considering the data source, the accuracy of the results, and other factors, we chose the industry head platform MOOC network as an example. Through the exploration of the MOOC teaching model of basic education and based on the fuzzy decision tree algorithm, we establish the member experience evaluation model. Finally, experiments show that the accuracy of the model is good and the reliability of the prediction results is high. However, there are many online Internet platforms in China, and with the further development of online video teaching industry, the characteristics of each platform are also different. Therefore, whether the fuzzy decision tree model proposed in this paper can be applied to other online basic education models remains to be discussed. The research scope can be further expanded in future work.

## Data Availability

The data used to support the findings of this study are available from the corresponding authors upon request.
